# Design of Thermochromic Polynorbornene Bearing Spiropyran Chromophore Moieties: Synthesis, Thermal Behavior and Dielectric Barrier Discharge Plasma Treatment

**DOI:** 10.3390/polym9110630

**Published:** 2017-11-22

**Authors:** Saleh A. Ahmed, Rawda M. Okasha, Khalid S. Khairou, Tarek H. Afifi, Abdel-Aleam H. Mohamed, Alaa S. Abd-El-Aziz

**Affiliations:** 1 Chemistry Department, Faculty of Applied Sciences, Umm Al-Qura University, Makkah 21955, Saudi Arabia; 2 Chemistry Department, Faculty of Science, Assiut University, Assiut 71516, Egypt; kskhairou@uqu.edu.sa; 3 Chemistry Department, Faculty of Science, Taibah University, Al-Madinah Al-Munawarah 30002, Saudi Arabia; rokasha@taibahu.edu.sa (R.M.O.); tmahmoud@taibahu.edu.sa (T.H.A.); 4 Physics Department, Faculty of Science, Taibah University, Al-Madinah Al-Munawarah 30002, Saudi Arabia; amohammedb@taibahu.edu.sa; 5 Physics Department, Faculty of Science, Beni-Suef University, Beni-Suef 62511, Egypt; 6 Chemistry Department, Faculty of Science, University of Prince Edward Island, Charlottetown, PE C1A 4P, Canada; abdelaziz@upei.ca

**Keywords:** polynorbornene, thermochromic, spiropyran, J-aggregation, ROMP, DBD plasma, SEM images

## Abstract

A new class of thermochromic polynorbornene with pendent spiropyran moieties has been synthesized. Functionalization of norbornene monomers with spirobenzopyran moieties has been achieved using Steglich esterification. These new monomeric materials were polymerized via Ring Opening Metathesis Polymerization (ROMP). In spite of their poor solubility, polynorbornenes with spirobenzopyran exhibited thermochromic behavior due to the conversion of their closed spiropyran moieties to the open merocyanine form. Moreover, these polymers displayed bathochromic shifts in their optical response, which was attributed to the J-aggregation of the attached merocyanine moieties that were associated with their high concentration in the polymeric chain. The surface of the obtained polymers was exposed to atmospheric pressure air Dielectric Barrier Discharge (DBD) plasma system, which resulted in the reduction of the surface porosity and converted some surface area into completely non-porous regions. Moreover, the plasma system created some areas with highly ordered J-aggregates of the merocyanine form in thread-like structures. This modification of the polymers’ morphology may alter their applications and allow for these materials to be potential candidates for new applications, such as non-porous membranes for reverse osmosis, nanofiltration, or molecular separation in the gas phase.

## 1. Introduction

Chromogenic polymers are a significant class of smart materials that have been under investigation over the past decade [1,2,3,4,5,6]. The key feature of these materials is their quick and reversible responses to external stimuli. Among these chromogenic materials, photochromic and thermochromic behavior has been observed in various classes of molecules, such as liquid crystals and polymer incorporating smart moieties [1,2,3,4,5,6]. Currently, there is progress in the development of thermochromic polymers as promising candidates for emerging areas, such as smart windows, biological sensors, and intelligent packaging.

The thermochromic effect can be introduced to the polymeric systems via incorporation of thermochromic leuco dye into the polymer chains [7,8]. Examples of these dyes include spirolactones, fluorans, spirooxazine, and spiropyran leuco dyes [1,3,4,5,9]. Spiropyran, as a family of photochromic and thermochromic materials, have been known for their inter-conversion from a closed form (SP) into an open merocyanine (MC) form upon irradiation with UV-light. There are advantages of these molecules that include high efficiency of photoisomerizations, sufficient thermal stability of both the open and the closed forms, and a very high resistance to photofatigue [10,11,12,13,14,15,16,17,18]. Spiropyran also has an impressive capability of isomerization by several other independent stimuli, such as temperature [10] (thermochromism), pH [11,12,13] (acidochromism), solvent polarity [14] (solvatochromism), redox potential [15] (electrochromism), metal ions [16,17], and even mechanical force [18] (mechanochromism).

Spiropyran has been merged into different classes of polymeric materials. For instance, homopolymers, random copolymers, and block copolymers containing spiropyran were synthesized via polymerization of monomeric units containing spiropyran motif using ring-opening metathesis polymerisation (ROMP) [19,20], AIBN-initiated free radical polymerisation of terminal alkenes [21,22,23,24,25,26,27], or by polycondensation reactions [28,29,30,31] and atom transfer radical polymerization (ATRP) [32,33,34] or reversible addition–fragmentation transfer (RAFT) polymerization [35,36,37]. Meanwhile, spiropyran has also been grafted on pre-formed polymers like polytetrafluoroethylene (PTFE) [38], polyaniline [39], polyacrylates [40,41,42], polysulfones [43,44], and polyphosphazenes [45].

Even though there are few examples of spiropyran functionalized polynorbornene reported in the literature as photochromic macromolecules [19,20], this class of macromolecules still requires further investigation in order to have a better understanding of the influence of the spiropyran moiety on the performance of the polymer chains, especially as thermochromic materials. For instance, Hauser et al. reported the synthesis of photochromic norbornene homo- and copolymers with spiropyran moieties [19]. The authors faced some difficulties in obtaining the homopolymer in good yields due to the formation of the opened merocyanine form. On the other hand, their copolymers were quite successful [19]. Unlike all of the previous reports that illustrated the synthesis of polynorbornene containing spiropyran as photochromic systems [19,20], this work is the first example of exploring the thermochromic behavior of polynorbornene bearing spiropyran switches.

Ring opening metathesis polymerization (ROMP) of norbornene monomers has been a very successful approach to control the polymer’s molecular structure, size, and bulk properties [46,47,48,49,50,51,52,53]. In the past, we have used Grubbs catalyst in the synthesis of various substituted polynorbornenes [54,55,56,57,58,59,60,61]. We also developed several series of polynorbornene containing aryl- and hetaryl-azo chromophores as candidates for long-term storage information, as well as in photonic devices [62]. Our previous work has also been dedicated to the synthesis of various classes of chromogenic molecules containing a spiro carbon, such as benzopyrans, oxazines, and dihydroindolizines, which are appropriate chemosensors that respond to external stimuli, including temperature [63,64,65,66,67,68,69,70].

This article will shed more light on the synthesis, characterization, and unexpected thermochromic properties of polynorbornenes containing spiropyran species using ROMP. These polymeric materials displayed thermochromic behavior due to the cleavage of the spiro C–O bond and forming the zwitterionic merocyanine form. This class of polymers is well known for its high surface porosity, which suits plenty of applications, such as smart windows, tunable light filters, large-area displays, as well as sensors that can visualize local surface temperatures [19,20,71].

Modification of a polymer’s morphology is a key feature of controlling its properties while maintaining its adequate bulk properties [72,73,74,75]. Surface modification of polymeric materials is one of the most attractive topics in the literature, which conjures the possibilities of a new era of applications and enhances the performance of these materials. Modification of the polymeric surface can be obtained using various approaches, such as flame, corona discharge, plasma, photons, electron beam, ion beam, X-ray, and γ-ray [72,73,74,75]. The use of a plasma system is a versatile and successful strategy for modifying the surface properties or introducing desired chemical groups at the surface of a polymer without affecting its bulk properties [72,73,74,75]. In this study, the surface of the obtained polymers has been subjected to an atmospheric pressure air Dielectric Barrier Discharge (DBD) plasma system in order to examine its effects on the polymers’ morphology. Modification of the surface character of the desired polymers allowed for these materials to be potential candidates for new applications, such as non-porous membranes for reverse osmosis, nanofiltration, or molecular separation in the gas phase.

## 2. Materials and Methods

### 2.1. Materials and Characterization

#### 2.1.1. General

All of the chemicals were available from Sigma-Aldrich Chemical Co. (Taufkirchen, Germany) and were used without further purification. Solvents were high performance liquid chromatography (HPLC) grade. ROMP was performed in anhydrous dichlormethane (DCM) ˃99.8% containing amylene as stabilizer. ^1^H NMR and ^13^C NMR spectra were recorded at 400 and 100 MHz, respectively, on Bruker 400 MHz ultrashieldTM NMR spectrometer (Bruker, Fällanden, Switzerland), with chemical shifts calculated in Hz, referenced to solvent residues. The IR spectra of the new compounds were recorded on a Jasco FTIR-300 E Fourier Transform Infrared Spectrometer (Jasco, Tokoyo, Japan). Thermogravimetric analysis (TGA) of the polymeric materials was carried out under nitrogen atmosphere at a heating rate 10 °C·min^−1^ using TA instrument, model SDT600 (New Castle, DE, USA). Differential Scanning Calorimeter (DSC) measurements were performed using a q2000 apparatus with T zero^®^ techniques from TA instruments (New Castle, DE, USA). The morphological features of the polymers were determined using a “Shimadzu XRD-6000” computerized diffractometer (Shimadzu, Tokoyo, Japan), which consists of α-pw1400/90 stabilized X-ray generator, α-pw1050/70 vertical goniometer, α-pw1995/60 proportional counter, and α-pw1930 electronic panel. Nickel-filter copper radiation with λ = 1.541 Å was used in this investigation. Scanning Electron Microscope (SEM) measurements have been performed using SEM Model Philips XL 30 (FEI company, Hillsboro, OR, USA) with accelerating voltage 30 KV, magnification 10× up to 4000×, and resolution for W. The obtained polymers have been heated up to 180 °C and then cooled to room temperature either as dry or wet sheets. Wettability has been tested in dichloromethane, dimethyl formamide, methanol, chloroform, and aqueous solution.

#### 2.1.2. Dielectric Barrier Discharge (DBD) Plasma System

An atmospheric air-dielectric barrier discharge DBD system was used to generate atmospheric pressure plasma in air [76]. The plasma system consists of two parallel metallic electrodes (the copper cylinder and the stainless steel disc) separated by a 3 mm gape. The upper copper electrode has about a ~40 mm diameter and was covered on the bottom by a 2 mm thick dielectric quartz sheet 60 × 60 mm. A high voltage power-supply was connected to the upper electrode, while the lower electrode was grounded. The high voltage electrode was surrounded by a Teflon sleeve for operator safety. The power supply generated sinusoidal waveform signal of 23 kV and 25 kHz. The current and voltage waveforms were recorded using DPO7354 C −3.5 GHz-Tektronix oscilloscope (Tektronix Inc., Beaverton, OR, USA) with a person probe model: 6585 and P6015A −1:1000 Tektronix-high voltage probe, respectively. The treated samples were placed between the two electrodes on the lower stainless steel electrode. Plasma images were captured by Nikon digital camera D3200 (Nikon, Thailand) with AF-S Micro NIKKOR 105 mm lens.

### 2.2. Synthesis of the Target Spirobenzopyrans and Their Polymerization with Norbornene Moiety

#### 2.2.1. Synthesis of 1′,3′,3′-Trimethyl-6-nitrospiro[chromene-2,2′-indoline]-5′-carboxylic Acid (**4**)

In three necked flask connected with inlet dry argon, a mixture of 1,3,3-trimethyl-2-methylene-5-carboxy-3H-indole (1150 mg, 5 mmol) and 2-hydroxyl-5-nitrobenzaldehyde (970 mg, 6 mmol) in freshly distilled THF (100 mL) was heated under reflux and argon for 10 h. The mixture was cooled down in an ice bath, and the resulting precipitate was filtered off. The pure product was obtained after column chromatography on silica gel using DCM as eluent (*R*_f_ = 0.32) to yield the chromophore I as a pale yellow powder in (460 mg, 24%). ^1^H NMR (400 MHz, DMSO-*d*_6_) 1.13 (s, 3H, CH_3_), 1.24 (s, 3H, CH_3_), 2.76 (s, 3H, N–CH_3_), 6.02 (d, 1H, *J* = 10 Hz), 6.70 (d, 1H, *J* = 8 Hz), 6.92 (1H, d, 9 Hz), 7.26 (1H, d, 10 Hz), 7.69 (1H, d, 8 Hz), 7.81 (1H, dd, 2 and 8 Hz), 8.02 (1H, dd, 3 and 9 Hz), 8.24 (1H, d, 3 Hz), 12.39 (1H, s, COOH); ^13^C NMR (100 MHz, DMSO-*d*_6_) δ 19.9, 25.9, 28.9, 51.9, 106.4, 106.6, 116.0, 119.4, 121.4, 122.2, 123.4, 123.4, 126.4, 129.1, 131.4, 136.4, 141.3, 151.7, 159.4, 167.9.

#### 2.2.2. Synthesis of 3-(3′,3′-Dimethyl-6-nitrospiro[chromene-2,2′-indolin]-1′-yl)propanoic Acid (**5**)

Under an argon atmosphere, a mixture of 1-(2-carboxyethyl)-2,3,3-trimethyl-3H-indol-1-ium bromide (311 mg, 1 mmol) and (167 g, 1 mmol) 2-hydroxy-5-nitrobenzaldehyde was added to 100 mL dry ethyl methyl ketone in the presence of triethyl amine (1 mL), followed by heating under reflux in the dark for 24 h. After the removal of the solvent, the product was washed with ether and the pure product of chromophore **5** was obtained after column chromatography using ethyl acetate-cyclohexane (2:8 then 4:6) as eluent (*R*_f_ = 0.32). The resulting violet-red powder was recrystallized from ethanol to obtain 136 mg, for a 36.1% yield of yellow-brown powder. ^1^H NMR (400 MHz, DMSO-*d*_6_) 1.09 (s, 3H, CH_3_), 1.52 (s, 3H, CH_3_), 2.35 (m, 1H, CH_2_), 2.46 (m, 1H, CH_2_), 3.38 (m, 1H, N–CH_2_), 3.48 (m, 1H, N–CH_2_), 6.00 (d, 1H, *J* = 10 Hz), 6.63 (d, 1H, 10 Hz), 6.78 (t, 1H, *J* = 8 Hz), 6.86 (d, 1H, 10 Hz), 7.11 (m, 2H), 7.19 (d, 1H, 10 Hz), 7.99 (2H, dd, 3 and 9 Hz), 8.21 (d, 1H, 2 Hz); ^13^C NMR (100 MHz, DMSO-*d*_6_) δ 19.8, 26.8, 37.7, 44.29, 52.4, 106.9, 116.0, 118.9, 119.4, 120.9, 121.8, 122.4, 126.2, 127.4, 127.9, 134.2, 146.7, 160.4.

#### 2.2.3. Synthesis of Norbornene Monomers

*Exo*, *endo*-5-norbornene-2-methanol (2 mmol), chromophores **4** & **5** (0.5 mmol), *N*,*N*-dimethylaminopyridine (244 mg, 2 mmol) and 8 mL of CH_2_Cl_2_ were placed in a 50 mL round bottom flask. Dicyclohexylcarbodiimide (413 mg, 2 mmol) was dissolved in 2 mL of CH_2_Cl_2_ and added to the stirring mixture at 0 °C. The reaction was carried out at room temperature under a nitrogen atmosphere for 18 h. After the removal of the solvent, the crude product was extracted with CH_2_Cl_2_, washed with water, and dried over MgSO_4_. The solvent was removed and the resulting precipitate was then purified using silica column with hexane/ethyl acetate (8:2 then 6:4) solution, (*R*_f_ = 0.45).

Monomer **7**: ^1^H NMR (400 MHz, acetone-*d*_6_): δ(ppm) = 0.54 (d, 1H, H3n(n)), 1.15 (s, 3H, CH_3_), 1.23 (s, 3H, CH_3_), 1.11–1.44 (m, 4H, H7a(n), H7a(x), H7s(x), H3x(x)), 1.66–1.83 (m, 4H, H2x, H7s(n), H3x(n), H3n(x)), 2.14 (br. s, 1H, H2n), 2.75 (s, 3H, N–CH_3_), 2.83–2.95 (m, 2H, H1x & H4n), 3.10–3.13 (m, 1H, H4x), 3.26–3.29 (m, 1H, H1x), 3.39–3.49 (m, 1H, H1n), 3.76 (t, 1H, nor-CH_2_O(n)), 3.94 (t, 1H, nor-CH_2_O(n)), 4.12 (t, 1H, nor-CH_2_O(x)), 4.30 (t, 1H, nor-CH_2_O(x)), 5.91 (br. s, 1H, H6(n)), 5.96 (br. s, 1H, H6(x)), 6.01 (d, *J* = 9.0 Hz, 1H, Ar), 6.06 (br. s, 1H, H5(n)), 6.16–6.18 (m, 1H, H5(x),), 6.69 (d, *J* = 9.0 Hz, 1H, Ar), 6.84 (d, *J* = 9.0 Hz, 1H, Ar), 7.24 (d, *J* = 9.0 Hz, 1H, Ar), 7.68 (s, 1H, Ar), 7.85 (d, *J* = 9.0 Hz, 1H, Ar), 7.99 (d, *J* = 9.0 Hz, 1H, Ar), 8.22 (s, 1H, Ar).

^13^C NMR (100 MHz, DMSO-*d*_6_) δ 19.83, 25.78, 29.01 (CH_3_), 28.75 (C3(n)), 29.55 (C3(x)), 41.39 (C2(n)), 41.55 (C2(x)), 41.88 (C4(x)), 42.11 (C4(n)), 43.28 (C1(x)), 43.58 (C1(n)), 44.90 (C7(x)), 49.34 (C7(n)), 51.93 (Ar-C), 64.90 (nor-CH_2_O(n)), 65.81 (nor-CH_2_O(x)), 106.24, 106.74, 115.76, 119.14, 121.11, 121.23, 123.09, 123.28, 126.18, 128.99, 131.17 (Ar-C & Ar-CH), 132.80 (C6(n)), 136.74 (C6(x)), 136.94 (C5(n)), 137.76 (C5(x)), 136.88, 141.13, 151.83, 159.30 (Ar-C), 166.06 (CO). IR (KBr): 1640 cm^−1^ (CO).

Monomer **8**: ^1^H NMR (400 MHz, acetone-*d*_6_): δ(ppm) = 0.36–0.39 (m, 1H, H3n(n)), 1.08 (s, 3H, CH_3_), 1.20 (s, 3H, CH_3_), 1.18–1.38 (m, 4H, H7a(n), H7a(x), H7s(x), H3x(x)), 1.66–1.79 (m, 4H, H2x, H7s(n), H3x(n), H3n(x)), 2.15 (br. s, 1H, H2n), 2.50 (m, 1H, CH_2_), 2.58–2.60 (m, 1H, H1x), 2.69 (m, 1H, CH_2_), 2.84 (br. s, 1H, H4n), 2.98 (t, 1H, nor-CH_2_O(n)), 3.13–3.16 (m, 1H, H4x), 3.22 (m, 1H, N–CH_2_), 3.38 (m, 4H, N-CH_2_ & nor-CH_2_O(n), (x) & (x)), 3.30 (br. s, 1H, H1x), 3.51–3.56 (m, 1H, H1n), 5.87 (br. s, 1H, Ar), 5.89 (br. s, 1H, H6(n)), 5.97 (br. s, 1H, H6(x)), 6.01 (br. s, 1H, H5(n) & H5(x)), 6.56 (d, *J* = 9.0 Hz, 1H, Ar), 6.74–6.78 (m, 2H, Ar), 7.01–7.10 (m, 4H, Ar), 7.93 (d, *J* = 9.0 Hz, 1H, Ar), 8.05 (s, 1H, Ar).

^13^C NMR (100 MHz, DMSO-*d*_6_) δ 19.48, 25.65 (CH_3_), 29.19 (C3(n)), 31.41 (C3(x)), 39.46 (CH_2_), 41.05 (C2(n)), 41.33 (C2(x)), 41.42 (C4(x)), 41.75 (C4(n)), 41.93 (CH_2_), 42.88 (C1(x)), 43.19 (C1(n)), 46.01 (C7(x)), 48.98 (C7(n)), 52.33 (Ar-C), 64.79 (nor-CH_2_O(n)), 65.76 (nor-CH_2_O(x)), 106.33, 115.19 (Ar-CH), 118.56 (Ar-C), 119.13, 121.47, 121.53, 122.59, 125.42, 127.48, 128.13 (Ar-CH), 132.26 (C6(n)), 136.20 (C6(x)), 136.33 (C5(n)), 136.40 (C5(x)), 146.14, 159.03 (Ar-C), 168.54 (CO). IR (KBr): 1644 cm^−1^ (CO).

#### 2.2.4. Preparation of Polynorbornene Pendent Chromophore Units

In a 10 mL round bottom flask, the functionalized monomer and 0.5 mL of distilled dichloromethane for every 100 mg of monomer were combined and stirred under a nitrogen atmosphere. In a second flask, the catalyst ([M]/[I] = 100/1) was dissolved in an equivalent amount of dichloromethane and was also purged with nitrogen. The catalyst solution was then added drop wise to the stirring monomer solution, at which time a black sticky solid began to collect on the sides and bottom of the flask. The reaction was stirred for 1–4 h during which time more solid formed. The polymerization was then terminated by the addition of ethyl vinyl ether, and stirred for 15–30 min. The crude polymer was washed with dichloromethane to remove any remaining monomer. The purified polymer was then collected via suction filtration as a reddish sheet.

## 3. Results

### 3.1. Synthesis of the Chromophores

**Scheme 1:**
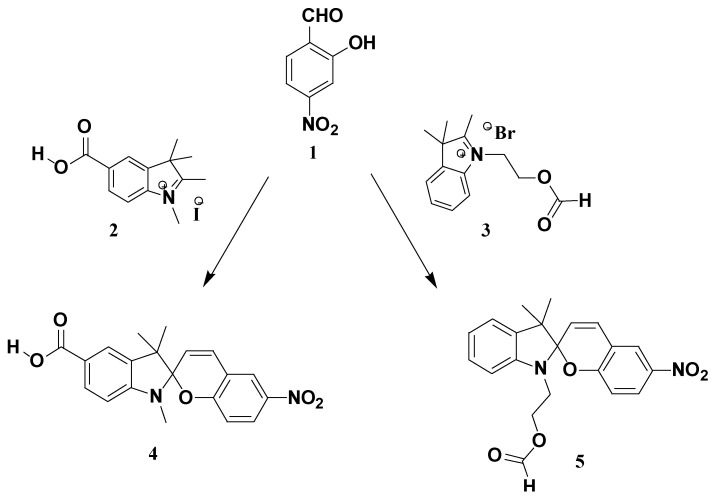
Synthesis of spiropyran chromophores.

The synthesis of compound **4** has been achieved via reaction of 2-hydroxyl-5-nitrobenzaldehyde **1** and 1,3,3-trimethyl-2-methylene-5-carboxy-3H-indole (**2**) in refluxed dry THF (Scheme 1). The crude product was isolated at 0 °C and purified through column chromatography using dichloromethane as eluent. Meanwhile, the condensation reaction of compound **1** with 1-(2-carboxyethyl)-2,3,3-trimethyl-3H-indol-1-ium bromide (**3**) and triethylamine in dry ethyl methyl ketone led to the formation of 3-(3′,3′-dimethyl-6-nitrospiro[chromene-2,2′-indolin]-1′-yl)propanoic acid (**5**) after further purification using column chromatography with ethyl acetate-cyclohexane as eluent.

The structure of chromophores **4** and **5** was confirmed using ^1^H and ^13^C NMR spectroscopy. As anticipated, the two spectra displayed some similarities. For example, the two methyl protons of both compounds appeared as singlet between 1.09 and 1.52 ppm, while the aromatic protons appeared as sets of doublets in the range of 6.00 and 8.24 ppm. Differentiation between the two spectra includes the presence of N–CH_3_ of chromophore **4** at 2.76 ppm, while chromophore **5** exhibited two sets of multiplets corresponding to the methylene spacers. The N–CH_2_ protons appeared as two multiplets at 3.38 and 3.48 ppm. Moreover, the carboxylic proton of **4** resonated downfield at 12.39 ppm.

### 3.2. Monomers Synthesis

**Scheme 2:**
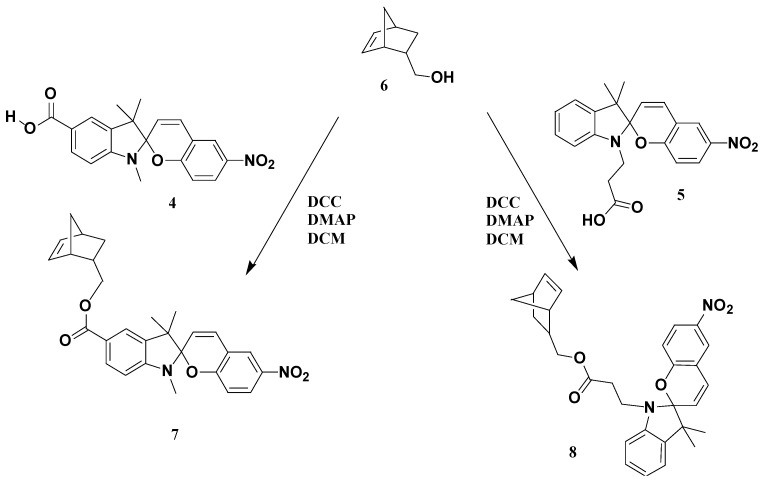
Synthesis of norbornene monomer functionalized with spiropyran moieties.

Functionalization of norbornene molecules with spiropyran chromophores was proceeded using Steglich esterification, as described in Scheme 2. *Exo, endo*-5-norbornene-2-mehanol (**6**) reacted with spiropyrans **4** and **5** in the presence of dicyclohexylcarbodiimide (DCC) and *N*,*N*-dimethylaminopyridine (DMAP) for 18 h. Purification of the obtained monomers included extraction with CH_2_Cl_2_, filtration of the dicyclohexylurea (DHU) side product, followed by purification over silica column using hexane/ethyl acetate solution.

The structural identity of the new monomeric units was accomplished using NMR spectroscopic analysis. For example, the carboxylic resonance at 12.38 ppm of spiropyran **4** disappeared from the corresponding monomer **7** due to the formation of the ester link. Also, the methylene protons of compound **5** was shifted further upfield upon esterification. Meanwhile, the presence of the new resonances of the norbornene moiety was observed. Since norbornene molecules exist as a mixture of their *exo* and *endo* isomers, their identity has been confirmed using two-dimensional (2D)-NMR spectroscopy. Identification of the *endo* isomer begins with the assignment of its olefinic protons, H_6_ and H_5_. These protons revealed strong coupling with each other and were assigned to the resonance in the range of 5.89–5.91 and 6.01–6.06 ppm for H_6_ and H_5_, respectively. The bridgehead protons, H_1_ and H_4_, were then recognized based on their correlations with the olefinic protons. H_1_ appeared between 3.39–3.49 ppm & 3.51–3.56 ppm for monomers **7** and **8**, respectively. Whereas H_4_ was found slightly upfield at 2.83–2.95 ppm for monomer **7** and at 2.84 for monomer **8**. The exclusive coupling of H_1_ and H_2_ in the *endo* isomer indicated that the broad singlet at 2.14 and 2.15 ppm is corresponding to H_2_n of monomer **7** and **8**, respectively. This assignment was verified based on the connectivity of the H_2_ resonance, with two peaks appearing further upfield in the spectrum in the range of 0.36–0.54 and 1.1.66–1.1.83 ppm, which represent the H_3*x*_ and the H_3*n*_, respectively. The bridge protons, H_7a_ and H_7s_, also exhibited strong coupling with each other and resonated as multiplets between 1.11–1.44 ppm (H_7a_) and 1.66–1.83 ppm (H_7s_).

The *exo* isomeric protons were identified using a similar stepwise strategy. The olefinic protons H_6_ and H_5_ appeared within the multiplet between 5.96–5.97 and 6.01–6.16 ppm for both isomers, while their characteristic couplings with the two bridgehead protons were then used to assign H_1_ to the multiplet in the range of 3.26–3.30 ppm and H_4_ to the broad singlet appearing between 3.10 and 3.16 ppm. In contrast to the *endo* isomer, H_2_ in the *exo* isomer does not have any connectivity with H_1_ [77,78]_,_ while the determination of this proton was based on its coupling with the two H_3_ protons, it was assigned to the multiplet between 1.66 and 1.83 ppm. H_3x_ (m, δ 1.11–1.44 ppm) was identified *via* its coupling with the bridgehead proton H_4_, however this coupling could not be observed with H_3n_ [77,78]. Finally, both H_7_ protons were assigned to the multiplet between 1.11 and 1.44 ppm.

### 3.3. ROMP and Thermal Analysis

**Scheme 3:**
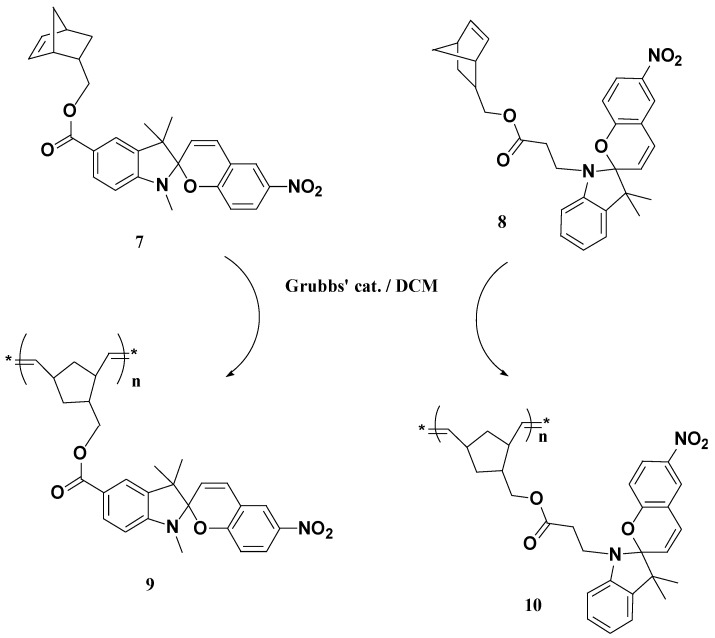
Ring opening metathesis polymerization (ROMP) of norbornene monomer functionalized with spiropyran moieties.

Polymerization of the new monomeric units, **7** and **8**, was achieved via ring opening metathesis polymerization (ROMP) using Grubbs’ catalyst. The reactions were performed using monomer/initiator ratio 100/1 over a period of 2 h. The resulting polymers precipitated from the dichloromethane solution during this time, and the reactions were terminated on the addition of ethyl vinyl ether, Scheme 3.

The new polymers were isolated as plastic sheets, which were completely insoluble in common organic solvents such as chloroform, dichloromethane, acetone, dimethylformamide, and dimethyl sulfoxide. The insolubility of these materials could be attributed to their high molecular weights along with the use of the low ratio of the initiator. The thermal behavior of the desired polymeric materials was performed using thermal gravimetric analysis (TGA) and differential scanning calorimetry (DSC). The TGA thermograms of polymers **9** and **10** revealed two distinctive weight losses. The first weight loss is between 400 and 447 °C, and is attributed to the partial decomposition of the pendent spiropyran motifs with degradation of the polymer backbone, while the second weight loss is between 530 and 600 °C and is corresponding to the degradation of the remaining polymeric chains.

**Figure 1:**
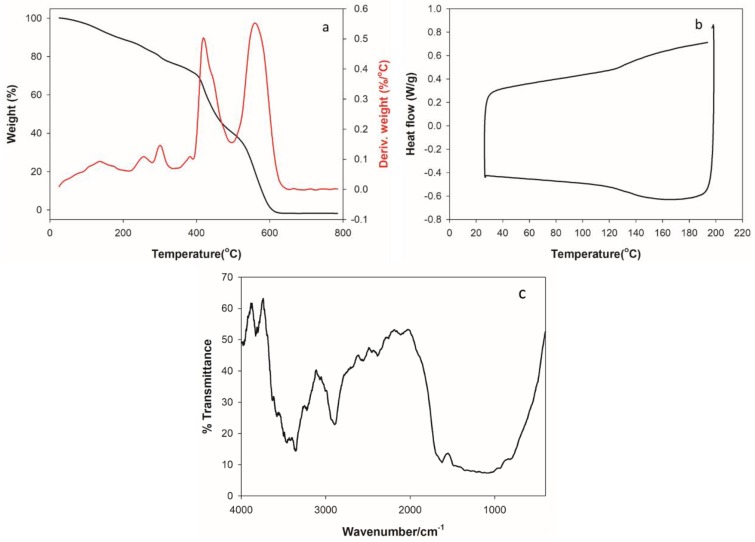
Thermogravimetric analysis (TGA) thermogram (**a**) and Differential Scanning Calorimeter (DSC) thermogram (**b**) and fourier transform infrared spectroscopy (FT-IR) spectrum (**c**) of polymer **9**.

The glass transition temperature of the new polymers was investigated using DSC measurements. It is well known that polynorbornene is a rather flexible chain, arising from its low *T*_g_ value [79,80]. This value can be manipulated via incorporation of different molecules into the side chain of the polymers [46,47,48,49,50,51,52,53,54,55,56,57,58,59,60,61,62]. Therefore, it is anticipated that the functionalization of norbornene monomers with the spiropyran moieties increases the *T*_g_ values of their corresponding polymers. The DSC thermograms of polymers **9** and **10** exhibits a *T*_g_ value of 136 °C and 127 °C, respectively. Figure 1 is representative examples of TGA, DSC thermograms, and FT-IR analysis.

### 3.4 Thermochromic Behavior

The prepared polymers displayed a reversible thermochromic behavior upon being exposed to heat either as a dry or wet solid sheet. The wettability was tested against humidity and in various organic solvents, such as dichloromethane, dimethyl formamide, methanol, and chloroform. Unlike some thermochromic systems [81], the new polymers displayed excellent thermochromic performance either as a dry or wet sheet. In general, this behavior corresponds to the transition process of the spiroheterocyclic moiety in the polymer side chain to the quasi-planar open merocyanine. The colored merocyanine form has been known for its strong tendency to associate into aggregates with a stack-like arrangement of the merocyanine molecules [82]. This behavior is triggered by the high concentration of the spiropyran molecules and manipulates their optical response [83,84]. The molecular dipole arrangement of the merocyanine usually takes place in two different styles, H-aggregates that are responsible for the production of the hypthochromic shift in the absorption band, while the J-aggregates produces the bathochromic shift in the absorption band [83,84].

**Figure 2:**
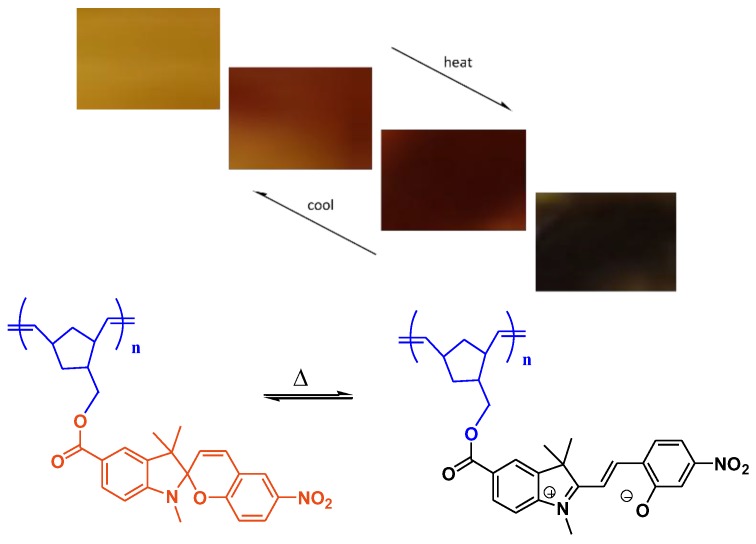
Thermochromic behavior of polymer **9**.

Figure 2 illustrates the influence of temperature on the polymer optical response. Upon exposure to heat, the color of the polymeric materials changed from reddish orange color into dark gray. This color range is attributed to the conformation of the spiropyran into the zwitrionic merocyanine form that is associated with bathochromic shift due to the presence of the J-aggregates.

### 3.5. Plasma Treatment and X-ray Diffraction

The surface properties of polymers are utilized for their possible applications, however these properties in most cases do not meet the required purposes. Tailoring the surface properties of the polymeric materials has been the focus of many studies [72,73,74,75]. One of the successful techniques that has been used to achieve this goal is the plasma treatment, which provides morphological changes to these materials [72,73,74,75]. Properties such as scratch-resistance, wettability, biocompatibility, gas transmission, adhesion, and friction could be optimized using different types of plasma systems, and afford great opportunities to suit the desired applications [72,73,74,75]. For example, Neves’ group reported the treatment of electrospun polycaprolactone nanofiber meshes (NFMs) by radio-frequency (RF) plasma containing (Ar or O_2_). This technique allowed for the improvement of the surface hydrophilicity through forming oxygen containing groups at the surface of the polymer, which enhanced their biological performance, such as adhesion and proliferation over three model cell lines, fibroblasts, chondrocytes, and osteoblasts [73].

**Figure 3:**
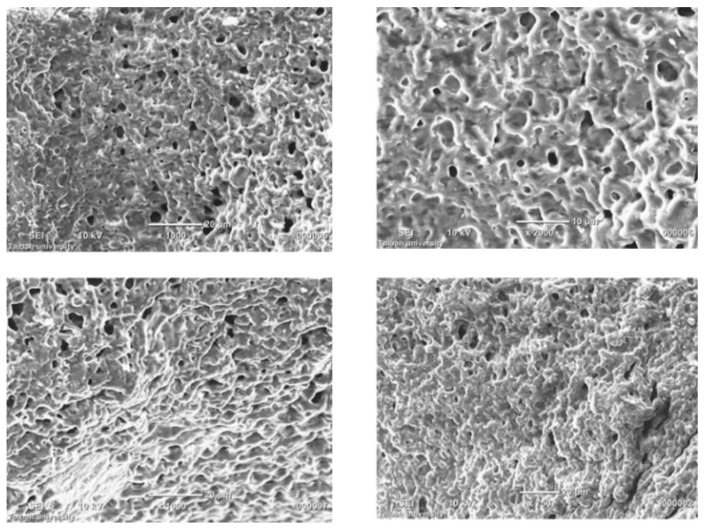
Scanning Electron Microscope (SEM) of polynorbornene containing spiropyran **9**.

**Figure 4:**
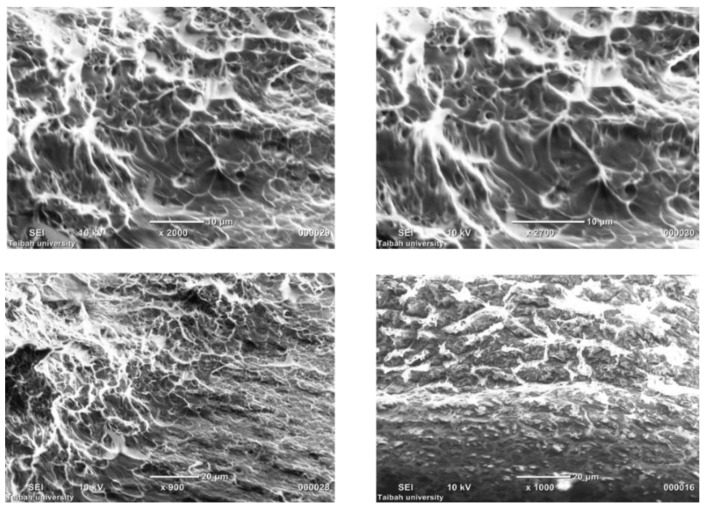
SEM of polynorbornene pendent spiropyran **9**. (After plasma treatment for 2 min (**top**) and 5 min (**bottom**)).

**Figure 5:**
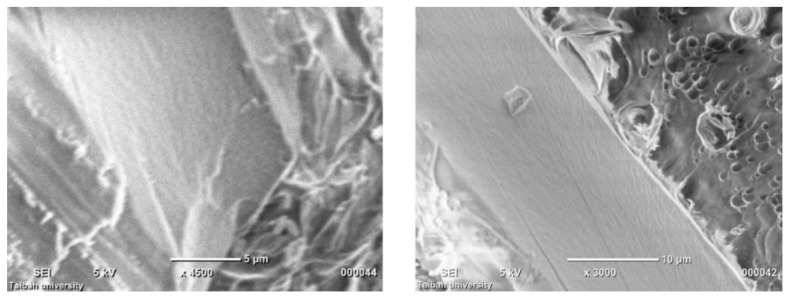
SEM of polynorbornene pendent spiropyran **9**. (After plasma treatment for 10 min).

In this study, the surface of the new polymers was examined using scanning electron microscope (SEM) in order to study its morphology. As anticipated, polynorbornene containing spiropyran molecules are distinguished by their highly porous and amorphous surface, as seen in Figure 3. Modification of the surface character has been achieved using atmospheric pressure air Dielectric Barrier Discharge (DBD) plasma treatment at different periods of time; the surface has been exposed to the plasma system for two, five, and ten minutes. The interaction with the surface was carried out using sinusoidal waveform signal of 23 kV and 25 kHz and its effect on the surface characteristic has been monitored via SEM images. As can be seen in Figure 4 and Figure 5, the porosity of the polymers has been reduced upon the plasma treatment. Moreover, with increasing the time period of the treatment, some surface areas have been changed to be completely nonporous. Other regions exhibited highly ordered thread-like structures that could be attributed to the J-aggregates of the merocyanine form [85]. These surface changes could broaden the applications of this class of materials into areas such as non-porous membranes for reverse osmosis, nanofiltration, or molecular separation in the gas phase.

**Figure 6:**
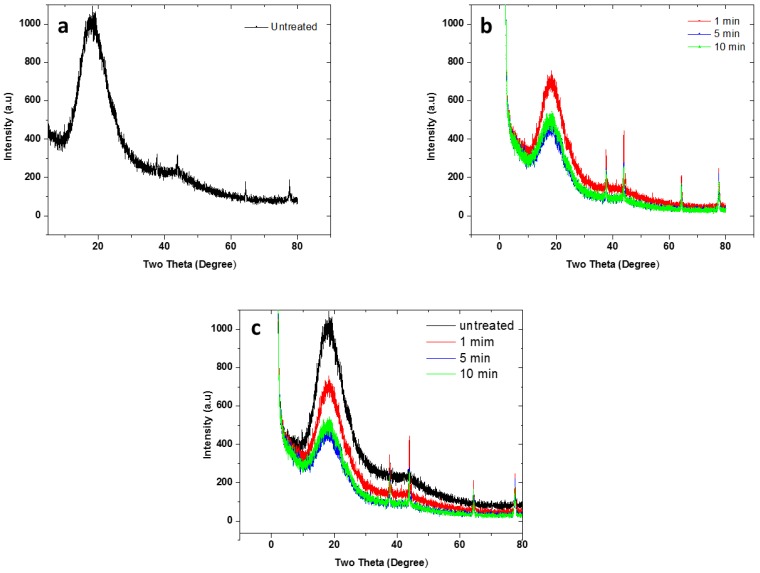
X-ray diffraction of polymer **9**. (**a**) the untreated sample; (**b**) after being exposed to the atmospheric pressure air Dielectric Barrier Discharge (DBD) plasma; (**c**) the untreated sample and the treated ones.

The X-ray diffraction of spiropyran containing polynorbornenes confirms the amorphous nature of the polymer, as seen in Figure 6a [86]. Moreover, the presence of the broad halo refers to the low stereoregularity of the polymer segments. The pattern shows a number of Bragg reflections, which can be indexed on the bases of the face centered cubic (FCC) structure of ruthenium. The diffraction peaks obtained at 2θ = 38.6, 44, 64.5, and 77.6′′ are identical with those reported for the standard ruthenium metal, which indicates the presence of some traces of ruthenium catalyst that have been trapped in the polymer matrix. Meanwhile, the interlayer spacing of polymer **9** (Figure 6a) can be calculated using the defined peak at 2Ɵ = 18.5° that is according to Bragg’s equation (with λ = 1.54 Å) and corresponds to d = 7.9 Å.

Figure 6b illustrates the diffraction patterns of polymer **9** after being exposed to the atmospheric pressure air Dielectric Barrier Discharge (DBD) plasma. As we mentioned previously, the treatment of the polymer surface with the DBD system has been tested at different periods of time. XRD comparison between the untreated sample and the treated ones (Figure 6c) showed that the width of the broad halo decreases with the increase of the treatment time. This could be attributed to the highly ordered J-aggregates of the merocyanine form in thread like-structures as a result of DBD plasma operating sinusoidal high voltage [85]. This hypothesis is also supported by the SEM images, Figure 5. It is also important to mention that the broad halo peaks for the treated polymers were not shifted to higher 2θ values, which means that the structures of these polymers did not change after the plasma treatment.

## 4. Conclusions

Chromogenic polymers hold a special place due to their potential applications in various fields, such as optical devices, data storage, and switches. This research work illustrates a successful attempt to functionalize norbornene monomers with spiropyran moieties to tailor the properties of their corresponding polymer. Successful ring opening metathesis polymerization of the new monomers allowed for the isolation of polynorbernene pendent spiropyran moieties. The new polymers displayed reversible thermochromic ability due to the conversion of the closed spiropyran moiety to the open merocyanine form that is associated with bathochromic shift due to the presence of the J-aggregates. In the meantime, the obtained polymers exhibited an amorphous nature with a high porosity. This texture enhances the thermochromic ability of the polymers due to the presence of the leuco dyes, as well as their nematic nature. Treatment of the obtained polymers with atmospheric pressure air Dielectric Barrier Discharge (DBD) plasma changes the porosity of the surface, which make this class of polymer eligible for various applications, such as non-porous membranes for reverse osmosis, nanofiltration, or molecular separation in the gas phase.
